# Program for Psychosocial Assessment During Pregnancy: Protocol for a Multimethod Study

**DOI:** 10.2196/67179

**Published:** 2025-08-01

**Authors:** Ylva-Li Lindahl, Helene Norén, Alkistis Skalkidou, Cecilia Åslund

**Affiliations:** 1 Centre for Clinical Research Uppsala University Västmanland Hospital Region Västmanland Västerås Sweden; 2 Department of Women´s and Children´s Health Faculty of Medicine Uppsala University Uppsala Sweden; 3 Child Health and Parenting (CHAP) Department of Public Health and Caring Sciences Faculty of Medicine Uppsala University Uppsala Sweden

**Keywords:** pregnant women, psychosocial intervention, obstetrics, perinatal mental health, midwifery, psychosocial, pregnant, pregnancy, maternal health care, maternal, Sweden, Europe, study protocol, mental health, mental illness, mental disorder, depression, questionnaire, interview, maternity, midwives

## Abstract

**Background:**

Onset of depression and anxiety can occur before, during, or after pregnancy, and ongoing conditions can either improve or deteriorate at all stages. National and international guidelines recommend that maternal health care should contribute to improving pregnant women’s mental health by consistently asking pertinent questions during pregnancy and offering necessary support and care. The implementation of a program for psychosocial assessment has been carried out in a region in Sweden. The program includes the identification of risk factors and ongoing mental ill-health, consultation with psychologists within maternal and child health care, and care pathways to other health care providers.

**Objective:**

The general aim of this research project is to investigate the effects of a program for psychosocial assessment during pregnancy on patients, as well as to evaluate the experiences of midwives and psychologists when working with the program.

**Methods:**

A multimethod study design will be used to evaluate possible effects of a program for psychosocial assessment during pregnancy, including a pre- and postimplementation comparison, alongside a qualitative study of the professionals’ perspectives. Data sources for the research project include patient data from the Swedish Pregnancy Register, medical records, questionnaire responses, and interviews with midwives and psychologists.

**Results:**

The recruitment process is completed, and data collection and analysis are ongoing. The dissemination plan includes several publications of original research and two doctoral theses.

**Conclusions:**

This study's results are expected to be relevant for maternal health care, both nationally and internationally, as scientific evaluations of the effects of implementing a program for psychosocial assessment during pregnancy are scarce. The implementation and evaluation of the program may hopefully, in the future, serve as a model for more standardized and effective care. It may influence national policies and attract attention as a model for improving perinatal mental health and maternity care.

**International Registered Report Identifier (IRRID):**

DERR1-10.2196/67179

## Introduction

### Background

This paper describes the study protocol, implementation, and planned evaluation of a program for psychosocial assessment during pregnancy in the Västmanland Region of Sweden.

In this protocol, mental ill-health is defined, according to the Public Health Authority of Sweden, as everything ranging from temporary anxiety, depression, or insomnia to severe mental illness. In order to be designated as mental ill-health, the symptoms must affect well-being and functionality in everyday life. Bipolar disorder, psychosis, severe depression, severe anxiety, and eating disorders are examples of severe mental illness [1].

The words “woman/women” will be used in this protocol to refer to pregnant people, in line with previous literature in the field. Although pregnancy is a physiological condition, rather than an illness, the word “patient” will also be used, since the subject relates to ill-health and illness.

During pregnancy, psychological symptoms are often normal and transient reactions to a stressful life situation. Mental ill-health during this life stage includes a spectrum of different conditions of varying severity [2]. A systematic review conducted in 2017 showed an overall prevalence of perinatal depression of 11.9% [3]. A study from Sweden found that 7% of women had major depression and 11% had an anxiety disorder during pregnancy [4], while a study from Iceland showed that anxiety and combined depression and anxiety were more common than depression alone during pregnancy [5]. In a meta-analysis, approximately 7% to 9% of pregnant women were estimated to meet the criteria for both depression and some form of anxiety [6]. Statistics from 2023 in the Swedish Pregnancy Register (SPR) showed that 11% of pregnant women in Sweden had been treated for mental ill-health [7].

Assessment of mental health in pregnancy is vital [8] and may, if undetected or unaddressed, affect the health of the woman, baby, and family [2]. Ongoing moderate to severe mental illness during pregnancy increase the risk for preterm birth, neonatal complications, and small-for-gestational-age babies [9]. Children of women with untreated or incompletely treated mental illness during pregnancy are at increased risk of emotional and behavioral problems while growing up [10].

Although many depression and anxiety conditions diagnosed in the postpartum period emerge during or before pregnancy [11], fewer women receive support and treatment for mental ill-health during pregnancy than in the postpartum period [12]. Women with prepregnancy moderate-to-severe mental illness are at increased risk of relapse during pregnancy and the initial postpartum period [12,13]. Therefore, it is important to identify these women early in pregnancy, particularly those with bipolar disorder and previous psychosis, because of the related increased risk of postpartum affective psychosis and other serious conditions. Early identification can be achieved by routinely asking all pregnant women structured questions, which enables the creation of a care plan for the pregnancy and postpartum period [11]. Early identification of depression and anxiety and timely treatment during pregnancy are essential and can reduce the rate of postpartum depression [11,14-17] as well as increase the possibility that the woman’s health will improve, enabling her to prepare for childbirth and motherhood [18].

A meta-analysis concluded that early identification, followed by thorough psychosocial and clinical assessment, is beneficial, even though evidence supporting a specific method remains limited [19]. At present, assessment with any tool can supplement a broader professional interview, to identify those requiring further clinical assessment and intervention. It is thus important that professionals have appropriate training and enough time to engage in a broad and meaningful conversation with the patient. Referral pathways must be created to ensure quick and accurate diagnostic assessment and effective interventions [17,19]. In Sweden, it has been recommended that a structured program to identify and treat mental health problems during pregnancy be created and that multidisciplinary perinatal mental health teams should be established in Sweden [20].

### Sweden’s Maternal Health Care System

The health care system in Sweden is primarily funded by regional governments through tax revenue. Residents have general access to publicly funded hospital care and primary health care, as well as maternal health care, child health care, and other publicly funded outpatient services.

There is regional autonomy, and organization varies; maternal health care can be run as part of primary care, as part of hospital obstetrics and gynecology departments, or within other organizations. However, regardless of region and organization, maternal health care coverage in Sweden is essentially 100%. Child health clinics in Sweden are responsible for child health surveillance from infancy to age 6 years. Similar to Swedish maternity clinics, they have essentially 100% coverage and are free of charge.

In Sweden, midwives, working in maternity clinics, are responsible for antenatal care, postpartum care, and sexual and reproductive health, collectively referred to as “maternal health care” in this paper. The maternal health care programs in Sweden are similar in terms of medical, obstetric, psychological, and social content, with some adaptations based on specific regional conditions. These programs usually comprise 9 to 10 visits during pregnancy and 2 visits after childbirth. In cases of comorbidity or complications, more visits can be offered. The midwife may consult, collaborate with, or refer to other professionals or agencies, such as a psychologist in maternal and child health care, general practitioner, obstetrician, psychiatrist, or social services. The midwife retains the main responsibility for care during collaborations of this type [21].

Psychologists within maternal and child health care in Sweden are employed at, or affiliated with, maternity clinics and child health clinics and will be referred to as “psychologists” in this paper. The psychologists have several professional functions, including consultation with midwives, most often in small groups, to obtain guidance for managing pregnant and postpartum women with manifest or suspected mental ill-health. The psychologists also assess and provide psychological treatment to individual women, co-parents and partners, and children for development assessment.

### Guidelines and Recommendations for Maternal Health Care in Sweden

Since 2015, the Swedish government has made major investments in improving perinatal health care and women’s health throughout life. All regions in Sweden are currently working to improve health care in various areas during the perinatal period [17,22].

Guidelines in Sweden from 2016 recommend that each woman be asked about her mental health in early pregnancy, but structured identification is not stipulated [21]. Furthermore, guidance concerning several aspects of maternity care (maternal health care, delivery, and postnatal care) has been subsequently published in 2022 and 2025 by the National Board of Health and Welfare [17,23]. Regarding perinatal mental health, the guidelines recommend that a structured and systematic approach with anamnesis of previous or ongoing moderate-to-severe mental illness in early pregnancy, including identification of depression and anxiety symptoms, should be used in maternal health care. The purpose is to enable preventive measures and early interventions, as well as in-depth assessment and diagnosis by a psychologist or physician, in order to address risks that may negatively affect parenting and thus the child.

According to the different guidelines, various implementations concerning antenatal and perinatal mental health have been established in different regions of Sweden. In Västmanland Region, a program for psychosocial assessment during pregnancy was implemented in spring 2020, according to national and international recommendations [16,21,24,25]. The purpose of the implementation was to provide women with appropriate interventions as early as possible during pregnancy, achieve equity in health care, decrease the postpartum depression rate, facilitate the midwives’ work, and develop collaborations between midwives and psychologists. The model used during the implementation process is closely aligned with the Quality Implementation Framework [26,27], and its phases correspond well to those applied in our implementation. The Quality Implementation Framework offers a comprehensive, structured approach for guiding the implementation process, with a focus on facilitating the translation of evidence-based practices into real-world settings [26]. In addition, our implementation process and associated strategies draw upon elements from other established process models and frameworks; however, the effectiveness or outcomes of the implementation itself will not be evaluated in this study.

Although guidelines have been published and various working methods have been implemented across Swedish regions, there is still a lack of evidence supporting a specific method, and no national consensus has been established regarding the most appropriate approach. Moreover, no scientific evaluation has yet been conducted to assess the effects of implementing a program to identify mental ill-health during the perinatal period in Sweden, despite its presumed importance for informing clinical practice. Although several countries have developed clinical guidelines for addressing perinatal mental health, their implementation and evaluation in practice remain insufficiently unexplored, both in Sweden and internationally. This limits our understanding of how to translate these guidelines into routine care and optimize outcomes for mothers, families, and health care systems. To address this knowledge gap, this project was initiated alongside the program’s implementation, to evaluate its effectiveness from both patient and professional perspectives.

### Aim

The general aim of this research project is to investigate the effects of a program in maternal health care for psychosocial assessment during pregnancy.

#### Primary Objectives

##### Objective 1

The first objective aims to answer: Does a program for psychosocial assessment during pregnancy influence the prevalence of pregnant women receiving psychological or medical treatment for mental ill-health?

##### Objective 2

The second objective aims to answer: Does a program for psychosocial assessment during pregnancy influence the time point for identification of mental ill-health in pregnant women?

#### Secondary Objectives

##### Objective 3

The third objective aims to answer: Does a program for psychosocial assessment during pregnancy influence the measures taken within the health care system and collaboration between the maternity clinic, perinatal psychologists, and other health care providers and agencies when mental ill-health is identified?

##### Objective 4

The fourth objective aims to answer: Does a program for psychosocial assessment during pregnancy influence women’s experiences with receiving help and support for identified mental ill-health?

##### Objective 5

The fifth objective aims to answer: What is the experience of midwives and psychologists when working with the program for psychosocial assessment during pregnancy?

## Methods

### Objectives

This study protocol outlines the full structure of an ongoing research program composed of multiple objectives, each at different stages of progress. Although certain elements—particularly parts of the data collection—have already been completed, others remain in the planning stage. The current status of each objective is as follows:

- Objectives 2-5: Participant recruitment has been completed.
- Objectives 1-3: Data collection is planned for 2025-2026, with results expected to be compiled in 2026.
- Objective 4: Data collection was completed in 2022; data analysis and results compilation are scheduled for 2025.
- Objective 5: Data collection was completed in 2024; analysis and results compilation are scheduled for 2025.

### Settings

The population of Sweden is approximately 10.5 million [28]. Västmanland Region, one of Sweden’s 21 regions, is of medium size with approximately 280,000 inhabitants and is located 108 km from Stockholm, the capital of Sweden. It is reasonably representative of Sweden in terms of the distribution of urban and rural areas, employment, income, education, and immigration levels. In 2023, there were 100,050 births in Sweden, of which 2568 occurred in the Västmanland Region [29].

In Västmanland Region, there are 26 maternity clinics spread out over the region. Approximately 55 midwives are employed; most clinics have 1 to 3 midwives, while 3 are staffed by 4 midwives. Since the implementation began, 3 clinics have closed, and 2 clinics have been added.

### Development and Implementation of a Program for Psychosocial Assessment During Pregnancy

The first author (YLL), a coordinating midwife and health care developer, and second author (HN), a psychologist specializing in perinatal mental health and health care developer, are both employed in the regional central maternal health care team and led the implementation and practical training for midwives. The implementation was conducted in collaboration with an associate professor and psychologist specializing in perinatal mental health.

#### Implementation Strategies

We involved 15 midwives and several psychologists in the design and adaptation of the program, as well as a pilot project to facilitate implementation in clinical practice. The program for psychosocial assessment was created concurring in content with, but actually preceding, the aforementioned national recommendations. The midwives’ experiences and opinions were recorded during meetings, and additional adjustments were made based on their suggestions for improvement. The adjustments were as follows: In addition to being asked in early pregnancy, all pregnant women are asked the questions about mental health (including the Whooley questions [30] and Generalized Anxiety Disorder 2-item [GAD-2] [31], see the following sections) again at gestational week 33. Those identified with mental ill-health and illness during pregnancy are asked again postpartum; in-depth questions concerning thoughts and feelings about childbirth, aimed at identifying fear of childbirth, are included; and the timing of questions concerning exposure to violence (already part of the health care program prior to the introduction of the program) was altered.

To carry out the implementation, the clinics’ funding was increased according to experiences in the pilot project. During the implementation, the need for contact with the psychologists also increased, and additional funding for psychological treatment was thus provided.

The program was developed, and supporting material was created to facilitate the midwives’ work. Collaborations between psychiatrists, obstetricians, general practitioners, and psychologists were established to clarify the referral pathways for women with identified mental illness or ill-health. Establishment of a multidisciplinary consulting team was reinforced with the aforementioned professionals, to provide guidance for the midwives regarding the management of the women identified with severe mental illness, as well as to strengthen the collaboration with other health care providers.

A central part of the implementation was to facilitate the midwives’ work and to ensure fidelity to the program. It was conducted with continual briefings in connection with their routine group consultations with the psychologists, and additional education and support were offered both individually and in small groups. To develop the consultation education, a consultation methodology took place at different levels for psychologists and midwives. Education concerning the program for psychosocial assessment was subsequently included in the mandatory introduction program for newly employed midwives.

#### Challenges in the Implementation

The maternity clinics in Västmanland Region are scattered throughout the county, and many clinics are quite small. As elsewhere in Sweden, there has also been a shortage of midwives in the region for several years. Combined with a vulnerable organization, this initially led to some difficulties in ensuring adherence and fidelity to the program, and implementation took longer than expected. A plan was conducted to facilitate the implementation process, with continuous information and education, support, and follow-up. The implementation process also took place during the COVID-19 pandemic, another aggravating circumstance.

#### Description of the Program for Psychosocial Assessment

**Figure 1:**
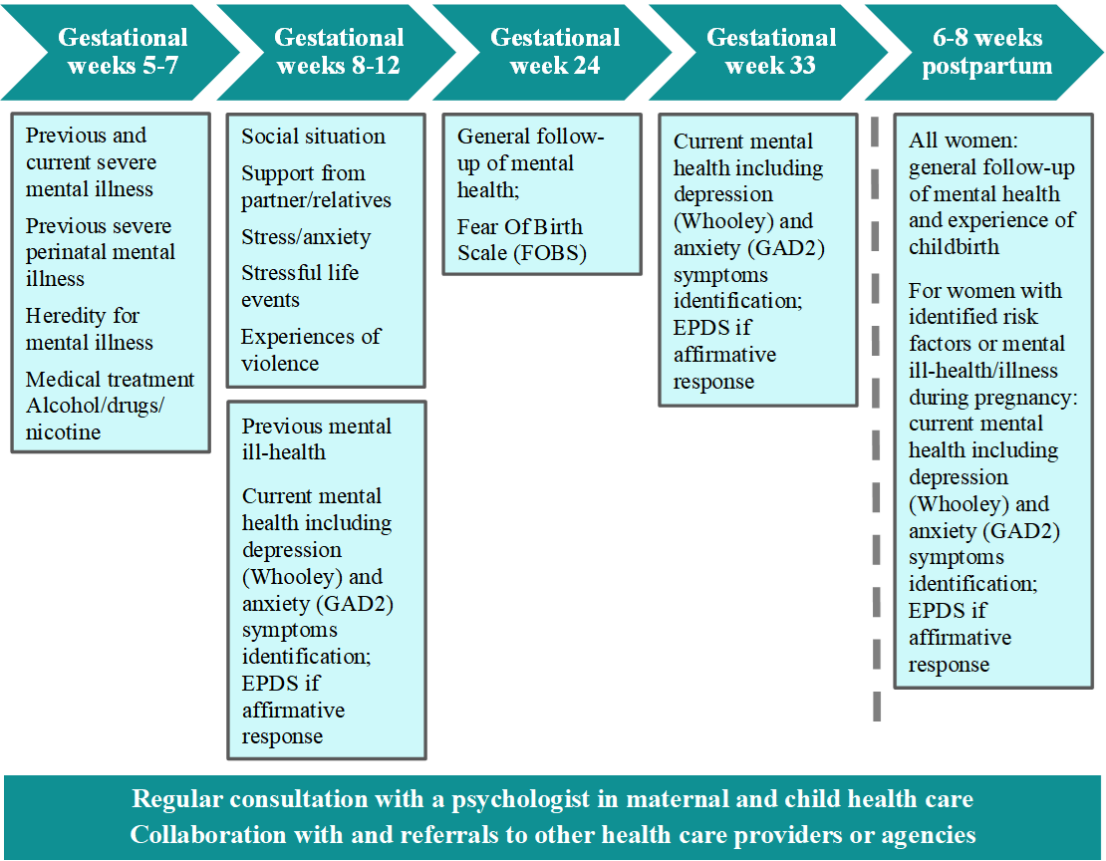
Overview of the program for psychosocial assessment (Västmanland Region), including maternity clinic visits and questions about mental health and social factors at various gestational weeks (+days); the visits after the dashed line (postpartum) will not be included in this evaluation. EPDS: Edinburgh Postnatal Depression Scale; GAD-2: Generalized Anxiety Disorder 2-item.

The program for psychosocial assessment comprises several components and is integrated into the overall maternity health care program for pregnancy monitoring in the Västmanland Region. It also includes consultation with psychologists and referral pathways to other health care providers. An overview of the program for psychosocial assessment is presented in Figure 1. The maternal health care program as a whole comprises 9 to 10 antenatal visits, ensuring comprehensive monitoring and support throughout pregnancy. The postpartum assessments (shown after the dashed line in Figure 1) will not be included in this evaluation.

At the first visit (gestational weeks 5-7), the midwife asks all women questions about prior and ongoing serious mental illness or perinatal mental illness and treatment, as well as family history of serious mental illness or perinatal mental illness. At the second visit, which takes place a few weeks later (gestational weeks 8-12), questions are asked concerning prior and ongoing less serious mental ill-health, symptoms of stress and anxiety, social situation, support from relatives and network, and experiences with domestic violence.

Moreover, to specifically identify symptoms of depression and anxiety during the conversation about mental ill-health, the midwife asks all women the Whooley questions [30] and GAD-2 questions [31].

The Whooley questions are designed to identify symptoms that may be present in depression. The questions are as follows:

- During the past month, have you often been bothered by feeling down, depressed, or hopeless?
- During the past month, have you often been bothered by little interest or pleasure in doing things?

The GAD-2 is a brief initial screening tool aimed at identifying symptoms of anxiety. The questions are as follows:

- Over the last two weeks, how often have you been bothered by feeling nervous, anxious, or on edge?
- Over the last two weeks, how often have you been bothered by not being able to stop or control worrying?

If the woman responds in the affirmative to any of these 4 questions, the Edinburgh Postnatal Depression Scale (EPDS) [32] is administered to aid further assessment [2,21,24]. The EPDS is a comprehensive 10-item self-rated scale for identifying symptoms of depression [32]. It has been validated in Sweden for use during pregnancy [33].

The Fear of Birth Scale [34,35] is a self-rated scale administered at gestational week 24 to identify fear of birth. If needed, additional visits are offered for childbirth preparation. There is also the possibility of referral to the Obstetrics Department’s specialist unit for assessment and care planning with a midwife, obstetrician, and perinatal psychologist.

The program for psychosocial assessment also includes an action plan for those identified as having a mental illness, including collaboration with other relevant providers and agencies. Routine consultations with psychologists provide midwives support in their assessments and in clinical decisions around referral for further assessment and treatment.

As aforementioned, women for whom risk factors or manifest mental illness have been identified during pregnancy are asked the Whooley and GAD-2 questions again at the postpartum visit, 6 weeks to 8 weeks after birth. This somewhat overlaps with the program at Swedish child health clinics, according to which the nurses administer the EPDS as a general screening method for all women at approximately 8 weeks postpartum [36].

### Study Design

**Figure 2:**
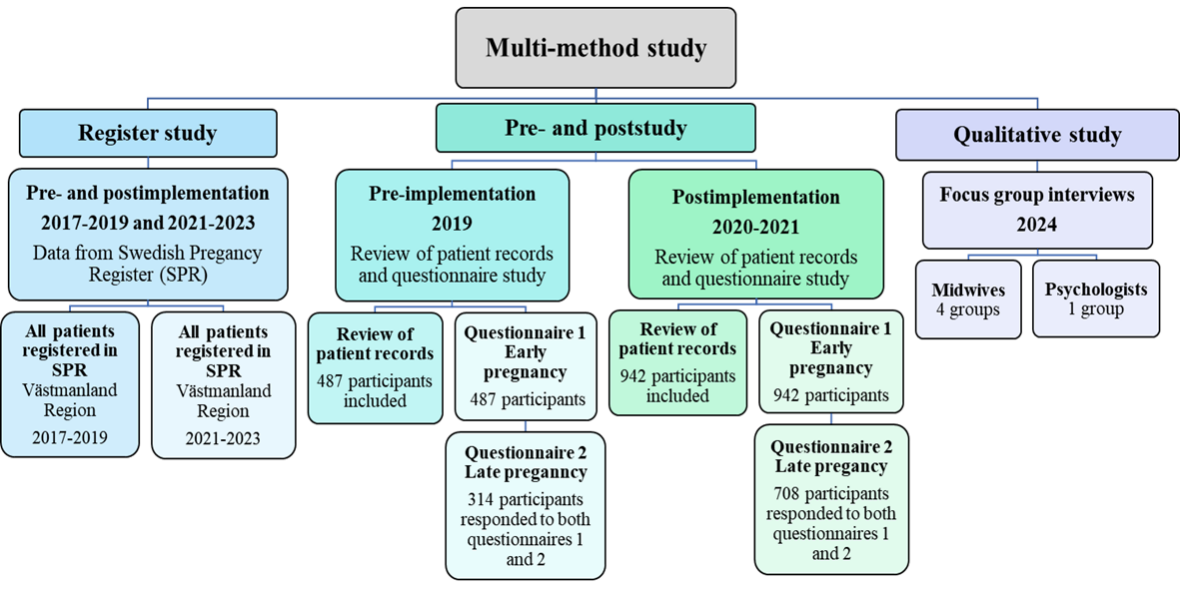
An overview of the research project, including study designs, data sources, included participants, and time periods.

The study will use a multimethod design incorporating both quantitative and qualitative research approaches. The quantitative part includes pre‑ and postimplementation assessments through the extraction of register-based data on all pregnant women in Västmanland Region during the defined pre‑ and postimplementation periods (objective 1). Furthermore, questionnaires and medical record data will be collected from study participants (objectives 2-4). The qualitative part will comprise focus group interviews with midwives and psychologists in maternal health care to explore their experiences with and perceptions of the implementation (objective 5). Detailed descriptions of the study design are provided in the corresponding sections for each objective. An overview of the study design is presented in Figure 2.

### Estimated Sample Size and Power

#### Sample Size Calculation

Calculation of the sample size was initially based on objective 1, using Minitab 16. The calculation was based on data from the SPR 2017 with a 7% initial prevalence of mental ill-health among pregnant women in the Västmanland Region [37], with an expected increase to 10% due to the rise in detection attributable to the program’s implementation. In order to detect an increase by 3 percentage points in identified mental ill-health, with 80% power and a significance level of 5% (2-sided test), at least 534 pregnant women had to be included in each group. The inclusion of several different maternity clinics in the project was also taken into consideration, and we estimated a dropout rate of approximately 20%. This meant that 640 pregnant women had to be included in each group for both pre- and postimplementation measurements.

However, the emergence of the COVID-19 pandemic introduced unforeseen challenges, particularly with regard to participant recruitment and data representativeness. It was hypothesized that the pandemic may have influenced the prevalence of mental ill-health in the target population, potentially confounding the original pre-post comparisons.

In response, a revised strategy for objective 1 was implemented. A new ethical approval was obtained to extend data inclusion and allow for the anonymous use of aggregated register data from the SPR, covering all pregnant women in the Västmanland Region during 2017-2019 (preimplementation) and 2021-2023 (postimplementation). This adjustment enabled a substantial increase in sample size and improved the generalizability of findings for objective 1 while minimizing selection bias and increasing statistical power.

Importantly, although the revised strategy for objective 1 now relies on registry-based data with a broader population base, the original sample size calculations remain valid for objectives 2-4, which continue to use the survey-based data set from pre- and postimplementation phases. The sample size for these objectives was not altered, and the statistical power for each is described in general terms in the following paragraphs.

Furthermore, addressing the recruitment challenges faced for objectives 2-4, it is crucial to note the difficulties encountered in achieving the initial target sample size. Specifically, we aimed to recruit 640 participants in the pre-implementation phase but were only able to enroll 487. This shortfall contrasted with the implementation phase, during which we successfully recruited 942 participants.

Given the research design outlined in the preceding paragraphs, together with the collected data, this will provide the opportunity to test a wide array of hypotheses in the current research area. Specifically, it is suitable for testing hypotheses involving both dichotomous outcomes, with lower or higher pre-implementation prevalence, and a wide range of continuous outcomes, including when testing for small, standardized differences.

#### Dichotomous Outcomes

The minimum detectable difference (MDD) with this design varies inversely with the pre-implementation prevalence. For instance, it can reliably, with 80% power, discern an 8% change in a 50% pre-implementation prevalence and more refined changes—such as 5% at a 10% prevalence or 4% at a 5% prevalence, with the same power at a higher prevalence (90% and 95%). This makes the design ideal for testing hypotheses expected to result in either a marked increase or decrease in prevalence rates, ensuring accurate detection of both common and rare conditions, as well as self-rated dichotomous factors according to the questionnaire data.

#### Continuous Outcomes

The study has the capacity to detect minimal standardized differences as small as 0.15 with 80% power. This level of sensitivity is particularly beneficial for hypotheses investigating subtle changes in continuous measures, such as gradual improvements or declines in health measures, psychological scores, continuous variables according to the questionnaire data, or any other continuous outcomes that fall well within the scope of this design. Given these conditions, the study design is suitable for a broad spectrum of hypotheses and can be used effectively to advance our knowledge in this field.

#### Handling of Missing Data

The proportion of missing data is expected to be low. Based on register quality and the data collection design, fewer than 5% of individuals are anticipated to have missing values for any variable included in the planned analyses. To assess whether the assumption of missing completely at random (MCAR) is valid, an MCAR test will be performed. This test examines whether the probability of missingness is independent of both observed and unobserved variables. If the assumption of MCAR is supported, complete-case analysis will be deemed appropriate. Should the test indicate that data are not MCAR, we will explore the pattern and mechanism of missingness further, applying multiple imputation or models to assess the robustness of results under plausible missing data scenarios. The final manuscripts will report the MCAR test results, the chosen method, and any observed differences in findings.

### Objective 1

#### Study Design

The study design incorporates pre- and poststudy measurements with comparison of pre- and postimplementation of a program for psychosocial assessment.

#### Participants and Data Collection

Data will be extracted from the SPR for all registered pregnant individuals in the Västmanland Region, preimplementation (2017-2019) and postimplementation (2021-2023) of the program. We chose 3-year periods for data extraction in order to obtain more representative data concerning any changes, as well as to counteract the effects of the COVID-19 pandemic.

The SPR is a national quality register with certification level 1 (the highest level). This accessibility is a prerequisite for the development of health care and consequently, for improving the health of pregnant women and their children, as well as promoting equitable care and high quality in the chain of care. Improvement work is facilitated by rapid feedback of SPR data and results. The SPR is a rich source for research in the field of pregnancy and childbirth. Of the 99,011 births registered in Sweden in 2023, 97,964 were registered in the SPR, a coverage rate of 98.9% [7].

#### Measurements

Objective 1 includes data from the SPR, which will be compared pre- and postimplementation of the program for psychosocial assessment, and captures the prevalence of patients who have received (1) medical treatment prepregnancy or during pregnancy, (2) psychological treatment for mental ill-health prepregnancy or during pregnancy, and (3) both medical and psychological treatment prepregnancy or during pregnancy.

#### Statistical Analysis

Logistic regression analysis, including an indicator variable for pre- and postimplementation, will be performed. The model will also include variables to adjust for potential confounding variables (eg, age, primiparous women, civil status, employment status, maternity clinic), selected according to the disjunctive cause criterion [38].

The effect measure will be the odds ratio (OR) with 95% CIs.

#### Statistical Power

Based on data from the SPR, which recorded 8246 births between 2017 and 2019 and 7611 births between 2021 and 2023, and an initial prevalence of identified cases of mental ill-health of 7%, the MDD for this register-based study was calculated. Using a 2-sided 2-sample *z* test for proportions with a 5% significance level (α=.05) and 80% power, the MDD was estimated at 0.8%. This indicates that the study will be capable of detecting changes of 0.8 percentage points or greater, thereby ensuring sufficient sensitivity to identify any clinically meaningful differences.

### Objective 2

#### Study Design

Pre- and poststudy assessments were conducted, with comparison of pre- and postimplementation of a program for psychosocial assessment.

#### Participants and Data Collection

Pregnant women who could communicate in Swedish and who were enrolled in early pregnancy at maternity clinics in the Västmanland Region were invited to participate in the study. In the poststudy, patients from all 25 maternity clinics in the region were invited to participate. The women were sent an envelope by post with an invitation to participate, a consent form, and a questionnaire. Consent covered the review of medical records (objectives 2 and 3) and questionnaire data (objective 4).

Participant inclusion was completed in 2022. Pre-implementation (2019), a request for participation was sent to 887 patients, and 487 were included. Questionnaire 2 was sent to 512 patients, and 314 responded to both questionnaires 1 and 2. Postimplementation (2020-2021), a request for participation was sent to 2368 patients, and 942 were included. Questionnaire 2 was sent to 837 patients, and 708 responded to both questionnaires 1 and 2.

A review of medical records will be conducted in the specific data system for antenatal and perinatal health care used in the region, to extract data for objective 2.

#### Measures

Objective 2 includes data extraction from medical records for the following variables: gestational age at identification of symptoms of mental ill-health and gestational age at diagnosis of mental ill-health.

#### Statistical Analysis

For objective 2, survival analysis will be used to assess differences in the timing of mental ill-health identification during pregnancy. Group comparisons will be performed using Cox proportional hazards regression analysis, including an indicator variable for pre- versus postimplementation. The model will be adjusted for potential confounders (eg, age, primiparous women, civil status, employment status, maternity clinic), selected according to the disjunctive cause criterion [38].

The effect measure will be the hazard ratio with 95% CIs.

#### Statistical Power

To evaluate the statistical power of this component of the study, we estimated the MDD based on the expected number of events. Drawing on the prevalence estimates and sample sizes outlined in the section “Estimated Sample Size and Power,” we approximated a total of 142 events across both study groups. Assuming a 2-sided significance level of 5% and 80% statistical power, the study is capable of detecting a hazard ratio of approximately 1.65, based on the method by Schoenfeld [39]. This suggests that the study is sufficiently powered to detect a 65% or greater difference in the rate of identification of mental ill-health between the pre- and postimplementation groups.

### Objective 3

#### Study Design

This pre- and poststudy design will compare the pre- and postimplementation of a program for psychosocial assessment.

#### Participants and Data Collection

Participant inclusion and data collection will be performed according to the description in objective 2.

#### Measures

Objective 3 includes data extraction from medical records for the following variables: whether the patient received psychological or medical treatment for mental ill-health that was initiated prepregnancy, whether the patient received psychological or medical treatment for mental ill-health that was initiated during pregnancy, and referral pathways to other health care providers when mental ill-health is identified.

#### Statistical Analysis

The proportion of patients referred to specific treatments before and after the intervention will be compared using logistic regression analysis. The primary focus will be on changes in referrals to psychological treatment. The regression model will include covariates selected based on the disjunctive cause criterion to adjust for potential confounding factors (eg, age, primiparous women, civil status, employment status, maternity clinic).

The effect measure will be the OR with 95% CIs.

#### Statistical Power

As described in the “Dichotomous Outcomes” subsection of the “Estimated Sample Size and Power” section, the study is powered to detect a change of approximately 4% to 5%, using a 2-sided test with an α level of 5% and 80% statistical power.

### Objective 4

#### Study Design

This pre- and poststudy design will compare pre- and postimplementation of a program for psychosocial assessment.

#### Participants and Data Collection

Regarding objective 4, participant inclusion and data collection have been completed, and data management and analysis are ongoing. Participant inclusion was performed according to the description in objective 2. In the 2 different patient groups, pre- and postimplementation questionnaires were sent twice during pregnancy. The first questionnaire was administered in early pregnancy after the first 2 visits to the midwife, and the second questionnaire was administered after gestational week 33.

#### Measures

The questionnaires covered the following areas: whether the midwife had asked questions about the participants’ mental health, their experience with the maternity clinic visits, whether they had been given support and help, previous psychiatric diagnoses, current mental health, treatment for mental ill-health during pregnancy, fear of childbirth, and self-assessed mental health.

Objective 4 will include questionnaire data for the following questionnaire items:

- How is your current mental health? (scale 1-10; 1=very poor, 10=very good)
- Have you had any psychological or psychiatric disorder diagnosed by a health care professional? (dichotomous response: Yes or No)
- To what extent do you feel that you received help for your mental health from your midwife? (scale 1-10; 1=not at all, 10=to a high degree)
- If you had extra visits with the midwife, to what extent do you feel they helped you? (scale 1-10: 1=not at all, 10=to a high degree)
- Did the midwife refer you to another health care provider? (dichotomous response: Yes or No)
- How long did it take to get in contact with another health care provider? (number of gestational weeks)

#### Statistical Analysis

Dichotomous outcomes will be analyzed using logistic regression models, while continuous outcomes will be analyzed using linear regression models. The regression model will include covariates selected based on the disjunctive cause criterion, to adjust for potential confounding factors (eg, age, primiparous women, employment status, maternity clinic).

For dichotomous outcomes, effects will be expressed as ORs with 95% CIs. For continuous outcomes, effects will be reported as mean differences, also with corresponding 95% CIs.

#### Statistical Power

As described in the “Estimated Sample Size and Power” section, the study is powered to detect a change of approximately 4% to 5% for dichotomous outcomes using a 2-sided test, with an α level of 5% and 80% statistical power. For continuous outcomes, the study is designed to detect standardized mean differences of approximately 0.15.

### Objective 5

#### Study Design

Focus group interviews will be conducted with an exploratory qualitative design and content analysis.

#### Participants and Data Collection

Regarding objective 5, participant inclusion and data collection have been completed, and analysis will be conducted in the next phase of the study. Participants are midwives who were working in the Västmanland region before, during, and after implementation of the program, as well as those who were employed after the implementation. We have conducted 4 focus group interviews with 3 to 5 midwives in each group, and we also included 1 group with 4 psychologists within maternal and child health care. The participants received verbal and written information and provided written consent.

#### Measures

Data were collected during semistructured focus group interviews based on an interview guide. Questions explore experiences with working with the program and whether the content of the consultation with psychologists and collaboration with other health care providers and agencies changed following the implementation of the program. The interviews were recorded and transcribed verbatim.

#### Data Analysis

Qualitative content analysis with an inductive approach, according to that by Graneheim and Lundman [40], will be undertaken [41,42]. The interviews will be analyzed by the authors. To obtain an overall impression of the data, the transcripts will be read repeatedly. Meaning units will be extracted, condensed, and coded according to relevance to the study aim. The codes will be sorted into subcategories with similar manifest content. The subcategories will be sorted into categories, followed by abstraction and interpretation of both manifest and latent content. The analysis process will be repeated several times and subsequently reviewed by one researcher with extensive experience in qualitative methods in order to ensure trustworthiness.

### Ethical Considerations

This project is carried out in accordance with the latest version of the Declaration of Helsinki and has been approved by the Swedish Ethical Review Authority (permit numbers 2018-331, 2018-332, 2019-02380, 2020-03479, 2020-06902, 2023-01892-02). All data will be managed and securely stored according to the General Data Protection Regulation (GDPR).

All collected materials, including interviews, questionnaires, and medical records will be treated with strict confidentiality and de-identified to protect participant privacy. Data management and storage at the Centre for Clinical Research in the Västmanland Region will strictly adhere to GDPR to ensure security.

Participants were provided information about the study, a consent form, and a questionnaire. Consent was requested to access data from the questionnaire and from medical records. Participants were informed that they had the right to withdraw from the study at any time. The completed questionnaires were sent directly to the Centre for Clinical Research in the Västmanland Region to ensure confidentiality. The review of medical records will be limited to the patient record system for pregnancy and delivery care, which is separate from the general medical records stored in the general system. All participants were informed that their responses would only be reported at a group level, ensuring individual confidentiality.

The participants received a gift card of Sk200 (US $20.91) following completion of the second questionnaire.

## Results

### Study Status

The recruitment of participants for objectives 2-5 is complete. Data collection for objectives 1-3 is planned for 2025-2026, with compilation of the results during 2026. Data collection for objective 4 was completed in 2022, and data analysis and compilation of the results are planned to be conducted in autumn 2025. Data collection for objective 5 was completed in 2024, and analysis and compilation of the results are also planned for autumn 2025.

The data collection for objectives 2-4 was extended in scope and time, as implementation took longer than estimated due, among other factors, to the COVID-19 pandemic outbreak in the first quarter of 2020 [43]. The pandemic was expected to create temporarily elevated levels of mental ill-health and illness, especially during pregnancy. Health care services in Sweden introduced restrictions for pregnant women’s partners accompanying them during and after pregnancy, which might have affected both expectant parents.

### Impact of Confounders and Contextual Factors

Several potential confounding factors must be acknowledged when interpreting the results of these future studies. The implementation was conducted within a fragmented organizational context, where variations between units in staffing, resources, and clinical priorities may have influenced both the fidelity of the program and its outcomes. In addition, the implementation coincided with the COVID‑19 pandemic, which introduced significant and unpredictable changes in clinical practice, patient flow, and staff availability. Importantly, the pandemic itself is likely to have influenced the measured outcomes, as it contributed to increased levels of anxiety, stress, and other forms of mental ill‑health among pregnant women. These factors may impact the effectiveness and the evaluation of the program, and the analysis of results must therefore be interpreted with these limitations in mind.

## Discussion

In this research project, we are in the process of investigating the effect of a program for psychosocial assessment during pregnancy.

### Principal Findings

This research project underscores the importance of early identification and treatment of mental health issues during pregnancy. Timely intervention can improve maternal mental health outcomes, including reducing the occurrence of postpartum depression [14,24]. This is crucial for the overall well-being of both the mother and the newborn. Furthermore, this research project explores midwives’ and psychologists’ experiences of working with the program, including consultation and collaboration with other health care providers and agencies following the implementation of the program. Improved collaboration and consultation can lead to a more comprehensive and coordinated approach, ensuring that pregnant women receive holistic care and support [17]. Another important aspect is the midwives’ and psychologists’ involvement in the evaluation process, allowing for insights into their experiences with the program and related training.

Research in the field of perinatal mental health can contribute to the ongoing development of maternal health care. The results of this research project are expected to be highly relevant for maternal health care, both in the national and international context and from several perspectives. The implementation of this program during pregnancy, as subsequently stipulated in the Swedish national recommendations, can serve as a model contributing to a more standardized and effective approach to maternal health care. If successful, the method might influence national guidelines and policies, setting a precedent for other regions and potentially attracting international attention as a model for improving maternity care. Our findings can further inform enhanced training and professional development programs, ensuring that health care professionals are well-equipped to implement programs for psychosocial assessment and provide high-quality and equitable maternal health care. 

### Comparison With Prior Work

Evidence on the effectiveness of screening and identification methods remains unclear, with variations across health care systems. In the United Kingdom [24] and Canada [44], a stepped approach is recommended, combining verbal questions with self-report scales. In the United States [45] and Australia [46], universal depression screening using the EPDS [30] is advocated, contingent on availability of diagnostic assessment, treatment, and follow-up. In Australia, psychosocial assessment is also recommended [47]. However, a recent Canadian review questioned the effectiveness of screening during pregnancy and postpartum when relying solely on an instrument [48].

### Strengths and Limitations

The research project includes comparison of the periods pre- and postimplementation. This is challenging, as the COVID-19 pandemic occurred in the early 2020s, in close proximity to the introduction of the program for psychosocial assessment. Our analytic strategy will need to include ways to account for the pandemic’s effects.

On the other hand, this project has many strengths, including the population-based design, uniformly designed educational programs for all midwives, and use of both quantitative and qualitative methods. Data sources include patient questionnaires, review of medical records, quality register data, and focus group interviews with midwives and psychologists. This multimethod design, combining pre- and poststudy assessment comparisons for patients and including the professionals’ perspective, is likely to enhance validity, depth, and interpretability. The findings will allow for both statistical evaluation of changes over time and a deeper understanding. This design is comprehensive and holistic, contributing to both theoretical and practical applications in the field.

No previous study has been conducted in Sweden to investigate the effect of a program of this type, and the results will thus be highly relevant for maternal health care in Sweden.

### Clinical Implications

Research in the field of perinatal mental health can contribute to the ongoing development of maternal health care. The results of this research project are expected to be highly relevant for maternal health care, both in the national and international context, and from several perspectives. The implementation of this program during pregnancy, as subsequently stipulated in the Swedish national recommendations, can serve as a model, contributing to a more standardized and effective approach to maternal health care. If successful, the method might influence national guidelines and policies, setting a precedent for other regions and potentially attracting international attention as a model for improving maternity care. Our findings can further inform enhanced training and professional development programs, ensuring that health care professionals are well-equipped to implement programs for psychosocial assessment and provide high-quality and equitable maternal health care. 

In summary, this research project is expected to contribute with a positive impact on the care chain in maternal health care by promoting early identification, improving support and treatment, implementing a structured approach, and fostering collaboration, to promote professional development and equitable health care. These outcomes collectively contribute to a more effective and patient-centered approach to maternal health care.

### Dissemination Plan

The dissemination plan encompasses continuous efforts toward the publication of several original research papers and the completion of two doctoral theses. It is hoped that this work will inspire further research, implementation, and evaluation of structured approaches addressing perinatal mental health.

### Future Directions

This research project has the potential to contribute to further studies within perinatal mental health and the application of interventions in clinical practice. However, there are several important questions that we will not be able to explore in depth and which we hope future research will address. An important knowledge gap is the investigation of the long-term effects of interventions in this area. Another important aspect is considering patients who do not speak Swedish—a group we excluded in this research project but one that is essential to evaluate in order to tailor methods and programs to their needs, particularly in relation to how cultural and contextual factors affect the implementation and outcomes of interventions. An additional area of interest is the development of theoretical models to better understand the mechanisms underlying observed effects, which could contribute to more effective interventions. Finally, it would be valuable to evaluate the implementation process itself and examine the effects of interventions under conditions without the presence of an ongoing COVID-19 pandemic, in order to understand how external factors influence the implementation process.

## Abbreviations

EPDS: Edinburgh Postnatal Depression Scale
GAD-2: Generalized Anxiety Disorder 2-item
GDPR: General Data Protection Regulation
MCAR: missing completely at random
MDD: minimum detectable difference
OR: odds ratio
SPR: Swedish Pregnancy Register

## Data Availability

The data sets generated or analyzed during this study cannot be publicly shared due to ethical or legal reasons but are available from the corresponding author on reasonable request.

## References

[1] The Public Health Agency of Sweden Vad är psykisk hälsa?. Folkhälsomyndigheten.

[2] Howard L, Molyneaux E, Dennis C, Rochat T, Stein A, Milgrom J (2014). Non-psychotic mental disorders in the perinatal period. The Lancet.

[3] Woody C, Ferrari A J, Siskind D J, Whiteford H A, Harris M G (2017). A systematic review and meta-regression of the prevalence and incidence of perinatal depression. J Affect Disord.

[4] Andersson L, Sundström-Poromaa Inger, Wulff Marianne, Aström Monica, Bixo Marie (2006). Depression and anxiety during pregnancy and six months postpartum: a follow-up study. Acta Obstet Gynecol Scand.

[5] Lydsdottir L, Howard Lm, Olafsdottir H, Thome M, Tyrfingsson P, Sigurdsson Jf (2014). The mental health characteristics of pregnant women with depressive symptoms identified by the Edinburgh Postnatal Depression Scale. J. Clin. Psychiatry.

[6] Falah-Hassani K, Shiri R, Dennis C (2017). The prevalence of antenatal and postnatal co-morbid anxiety and depression: a meta-analysis. Psychol. Med.

[7] The Swedish Pregnancy Register Graviditetsregistrets Årsrapport 2023. https://www.medscinet.com/GR/uploads/hemsida/Graviditetsregistrets%20Årsrapport%202023%20v%202.0.pdf.

[8] Rubertsson C, Waldenström U, Wickberg B, Rådestad I, Hildingsson I (2005). Depressive mood in early pregnancy and postpartum: prevalence and women at risk in a national Swedish sample. Journal of Reproductive and Infant Psychology.

[9] Stein A, Pearson RM, Goodman SH, Rapa E, Rahman A, McCallum M, Howard LM, Pariante CM (2014). Effects of perinatal mental disorders on the fetus and child. The Lancet.

[10] Stein A, Netsi E, Lawrence PJ, Granger C, Kempton C, Craske MG, Nickless A, Mollison J, Stewart DA, Rapa E, West V, Scerif G, Cooper PJ, Murray L (2018). Mitigating the effect of persistent postnatal depression on child outcomes through an intervention to treat depression and improve parenting: a randomised controlled trial. The Lancet Psychiatry.

[11] Wisner K, Sit Dorothy K Y, McShea Mary C, Rizzo David M, Zoretich Rebecca A, Hughes Carolyn L, Eng Heather F, Luther James F, Wisniewski Stephen R, Costantino Michelle L, Confer Andrea L, Moses-Kolko Eydie L, Famy Christopher S, Hanusa Barbara H (2013). Onset timing, thoughts of self-harm, and diagnoses in postpartum women with screen-positive depression findings. JAMA Psychiatry.

[12] Munk-Olsen T, Maegbaek M L, Johannsen B M, Liu X, Howard L M, di Florio A, Bergink V, Meltzer-Brody S (2016). Perinatal psychiatric episodes: a population-based study on treatment incidence and prevalence. Transl Psychiatry.

[13] Howard LM, Khalifeh H (2020). Perinatal mental health: a review of progress and challenges. World Psychiatry.

[14] Austin M (2004). Antenatal screening and early intervention for "perinatal" distress, depression and anxiety: where to from here?. Arch Womens Ment Health.

[15] Buist A, Barnett Bryanne E W, Milgrom Jeannette, Pope Sherryl, Condon John T, Ellwood David A, Boyce Phillip M, Austin Marie-Paule V, Hayes Barbara A (2002). To screen or not to screen--that is the question in perinatal depression. Med J Aust.

[16] Curry Susan J, Krist Alex H, Owens Douglas K, Barry Michael J, Caughey Aaron B, Davidson Karina W, Doubeni Chyke A, Epling John W, Grossman David C, Kemper Alex R, Kubik Martha, Landefeld C Seth, Mangione Carol M, Silverstein Michael, Simon Melissa A, Tseng Chien-Wen, Wong John B, US Preventive Services Task Force (2019). Interventions to prevent perinatal Depression: US Preventive Services Task Force recommendation statement. JAMA.

[17] The National Board of Health and Welfare (2022). Graviditet, förlossning och tiden efter – Nationellt kunskapsstöd för kontinuitet i vårdkedjan och vård på rätt nivå. Socialstyrelsen.

[18] Swedish Association of Obstetrics and Gynaecology (2009). Barnafödande och psykisk sjukdom.

[19] Rondung E, Massoudi P, Nieminen K, Wickberg B, Peira N, Silverstein R, Moberg K, Lundqvist M, Grundberg Å, Hultcrantz M (2024). Identification of depression and anxiety during pregnancy: a systematic review and meta-analysis of test accuracy. Acta Obstet Gynecol Scand.

[20] Wickberg B, Bendix M, Wetterholm MB, Skalkidou A (2020). Perinatal mental health around the world: priorities for research and service development in Sweden. BJPsych Int.

[21] Swedish Association of Obstetrics and Gynaecology, The Swedish Association of Midwives, The Swedish Association of Perinatal Psychologists (2016). Mödrahälsovård, sexuell och reproduktiv hälsa.

[22] Swedish Association of Local Authorities and Regions Kvinnors hälsa- Sveriges Kommuner och Regioner.

[23] The National Board of Health and Welfare Nationella riktlinjer 2025 – Graviditet, förlossning och tiden efter. Socialstyrelsen.

[24] National Institute for Health and Care Excellence (NICE) (2020). Antenatal and postnatal mental health: clinical management and service guidan.

[25] Research Innovation and Sustainable Pan-European Network in Peripartum Depression Disorder (Riseup-PPD) (2023). Evidence-Based Clinical Practice Guidelines For Prevention, Screening and Treatment Of Peripartum Depression.

[26] Meyers D, Durlak Joseph A, Wandersman Abraham (2012). The quality implementation framework: a synthesis of critical steps in the implementation process. Am J Community Psychol.

[27] Nilsen P (2015). Making sense of implementation theories, models and frameworks. Implement Sci.

[28] (2023). Statistics Sweden.

[29] Statistics Sweden (2023). Födda i Sverige 2023.

[30] Whooley M, Avins A L, Miranda J, Browner W S (1997). Case-finding instruments for depression. Two questions are as good as many. J Gen Intern Med.

[31] Kroenke K, Spitzer Robert L, Williams Janet B W, Monahan Patrick O, Löwe Bernd (2007). Anxiety disorders in primary care: prevalence, impairment, comorbidity, and detection. Ann Intern Med.

[32] Cox J, Holden J M, Sagovsky R (1987). Detection of postnatal depression. Development of the 10-item Edinburgh Postnatal Depression Scale. Br J Psychiatry.

[33] Rubertsson C, Börjesson Karin, Berglund Anna, Josefsson Ann, Sydsjö Gunilla (2011). The Swedish validation of Edinburgh Postnatal Depression Scale (EPDS) during pregnancy. Nord J Psychiatry.

[34] Haines H, Pallant Julie F, Karlström Annika, Hildingsson Ingegerd (2011). Cross-cultural comparison of levels of childbirth-related fear in an Australian and Swedish sample. Midwifery.

[35] Hildingsson I, Haines Helen, Karlström A, Nystedt A (2017). Presence and process of fear of birth during pregnancy-findings from a longitudinal cohort study. Women Birth.

[36] The National Handbook for Child Health Services Screening med EPDS - Rikshandboken i barnhälsovård.

[37] The Swedish Pregnancy Register Graviditetsregistrets Årsrapport 2019.

[38] VanderWeele T, Shpitser Ilya (2011). A new criterion for confounder selection. Biometrics.

[39] Schoenfeld D (1983). Sample-size formula for the proportional-hazards regression model. Biometrics.

[40] Graneheim U, Lundman B (2004). Qualitative content analysis in nursing research: concepts, procedures and measures to achieve trustworthiness. Nurse Educ Today.

[41] Graneheim U, Lindgren B, Lundman B (2017). Methodological challenges in qualitative content analysis: a discussion paper. Nurse Educ Today.

[42] Lindgren B, Lundman Berit, Graneheim Ulla H (2020). Abstraction and interpretation during the qualitative content analysis process. Int J Nurs Stud.

[43] World Health Organization (2020). WHO Director-General's opening remarks at the media briefing on COVID-19 - 11 March 2020.

[44] Provincial Council for Maternal and Child Health (2021). Perinatal Mental Health - Guidance for the identification and management of mental health in pregnant or postpartum individuals.

[45] Siu A, Bibbins-Domingo Kirsten, Grossman David C, Baumann Linda Ciofu, Davidson Karina W, Ebell Mark, García Francisco A R, Gillman Matthew, Herzstein Jessica, Kemper Alex R, Krist Alex H, Kurth Ann E, Owens Douglas K, Phillips William R, Phipps Maureen G, Pignone Michael P, US Preventive Services Task Force (USPSTF) (2016). Screening for Depression in Adults: US Preventive Services Task Force Recommendation Statement. JAMA.

[46] Austin MP, Highet N, Expert Working Group (2017). Mental Health Care in the Perinatal Period: Australian Clinical Practice Guideline. Central and Eastern Sydney Primary Health Network.

[47] Austin M, Colton Jana, Priest Susan, Reilly Nicole, Hadzi-Pavlovic Dusan (2013). The antenatal risk questionnaire (ANRQ): acceptability and use for psychosocial risk assessment in the maternity setting. Women Birth.

[48] Beck A, Hamel Candyce, Thuku Micere, Esmaeilisaraji Leila, Bennett Alexandria, Shaver Nicole, Skidmore Becky, Colman Ian, Grigoriadis Sophie, Nicholls Stuart Gordon, Potter Beth K, Ritchie Kerri, Vasa Priya, Shea Beverley J, Moher David, Little Julian, Stevens Adrienne (2022). Screening for depression among the general adult population and in women during pregnancy or the first-year postpartum: two systematic reviews to inform a guideline of the Canadian Task Force on Preventive Health Care. Syst Rev.

